# Metabolomic and Transcriptomic Analyses of Flavonoid Biosynthesis in Different Colors of Soybean Seed Coats

**DOI:** 10.3390/ijms26010294

**Published:** 2024-12-31

**Authors:** Yuanfang Fan, Sajad Hussain, Xianshu Wang, Mei Yang, Xiaojuan Zhong, Lei Tao, Jing Li, Yonghang Zhou, Chao Xiang

**Affiliations:** 1 Crop Research Institute, Sichuan Academy of Agricultural Sciences, Chengdu 610066, China; yuanfang518@scsaas.cn (Y.F.); wangxs1323690423@163.com (X.W.); yangmeitong2012@163.com (M.Y.); zhongxj@scsaas.cn (X.Z.); zyhmail2024@163.com (Y.Z.); 2 Environment-Friendly Crop Germplasm Innovation and Genetic Improvement Key Laboratory of Sichuan Province, Chengdu 610066, China; 3 College of Life Sciences, Shandong Agricultural University, Taian 271018, China; hussain.sajad@iub.edu.pk; 4 Sichuan Seed Station, Chengdu 610041, China; zzztaolei@163.com (L.T.); eteahenry@163.com (J.L.)

**Keywords:** soybean, flavonoid, transcriptome, metabolome, seed coat color

## Abstract

Soybean has outstanding nutritional and medicinal value because of its abundant protein, oil, and flavonoid contents. This crop has rich seed coat colors, such as yellow, green, black, brown, and red, as well as bicolor variants. However, there are limited reports on the synthesis of flavonoids in the soybean seed coats of different colors. Thus, the seed coat metabolomes and transcriptomes of five soybean germplasms with yellow (S141), red (S26), brown (S62), green (S100), and black (S124) seed coats were measured. In this study, 1645 metabolites were detected in the soybean seed coat, including 426 flavonoid compounds. The flavonoids differed among the different-colored seed coats of soybean germplasms, and flavonoids were distributed in all varieties. Procyanidins A1, B1, B6, C1, and B2, cyanidin 3-O-(6″-malonyl-arabinoside), petunidin 3-(6″-p-coumaryl-glucoside) 5-glucoside, and malvidin 3-laminaribioside were significantly upregulated in S26_vs._S141, S62_vs._S141, S100_vs._S141, and S124_vs._S141 groups, with a variation of 1.43–2.97 × 10^13^ in terms of fold. The differences in the contents of cyanidin 3-O-(6″-malonyl-arabinoside) and proanthocyanidin A1 relate to the seed coat color differences of red soybean. Malvidin 3-laminaribioside, petunidin 3-(6″-p-coumaryl-glucoside) 5-glucoside, cyanidin 3-O-(6″-malonyl-arabinoside), and proanthocyanidin A1 affect the color of black soybean. The difference in the contents of procyanidin B1 and malvidin 3-glucoside-4-vinylphenol might be related to the seed coat color differences of brown soybeans. Cyanidin 3-gentiobioside affects the color of green soybean. The metabolomic–transcriptomic combined analysis showed that flavonoid biosynthesis is the key synthesis pathway for soybean seed color formation. Transcriptome analysis revealed that the upregulation of most flavonoid biosynthesis genes was observed in all groups, except for S62_vs._S141, and promoted flavonoid accumulation. Furthermore, *CHS*, *CHI*, *DFR*, *FG3*, *ANR*, *FLS*, *LAR*, and *UGT88F4* exhibited differential expression in all groups. This study broadens our understanding of the metabolic and transcriptomic changes in soybean seed coats of different colors and provides new insights into developing bioactive substances from soybean seed coats.

## 1. Introduction

Soybean [*Glycine max* (Linn.) Merr.] is an essential nutritional and commercial crop that provides major plant proteins and oils for people and animals worldwide [1,2]. As a physical barrier, a soybean seed coat controls the metabolism of seed development and dormancy, disease resistance, and nutritional metabolism [3]. A soybean seed coat contains several active substances, such as dietary fiber, polyphenols, and flavonoids. These compounds are the focus of extensive research because of their remarkable antioxidant properties and ability to influence the flavor characteristics of the seeds [4]. In addition, these compounds play a crucial role in preventing cancer [5], inflammation, and cardiovascular diseases [6].

The soybean seed coat has evolved to be black, brown, green, and bicolor via artificial selection and domestication [7]. Flavonoids and anthocyanins are the main substances affecting the color of the soybean seed coat [8]. Flavonoids are widely distributed in higher plants, with flavones, chalcones, anthoxanthins, and proanthocyanidins present [9,10]. These substances play major roles in various biological processes, imparting various colors, antioxidant properties, and nutritional value [11,12]. Anthocyanins are important flavonoids that influence the coloration of the seed coat, which can cause plant organs to exhibit different color phenotypes [13]. Anthocyanins are synthesized through the flavonoid pathway and regulated by many structural genes, such as chalcone synthase (*CHS*), flavanone 3-hydroxylase (*F3H*), and anthocyanidin synthase (*ANS*) [12,14]. In soybean seeds, a large number of color changes and pigmentation patterns are observed [15].

Currently, 23 color-regulating genes have been reported for soybean seeds, and 19 genes are on the flavonoid synthesis pathway, such as *CHS*, chalcone isomerase (*CHI*), and UDP-glucose flavonoid 3-glucosyltransferase (*UFGT*) [16]. *CHS* regulates the content and activity of chalcone synthase, while the rate-limiting step governs the entire metabolic pathway. The transcription factor *MYB* at the R site regulates the content of anthocyanidin by controlling the content and activity of *ANS*, affecting the color of the soybean seed coat [17]. Anthocyanidins are catalyzed by anthocyanidin reductase (*ANR*) and UDP-glucose: flavonoid 3-O-glucosyltransferase (*UF3GT*), forming proanthocyanins and anthocyanins [7]. The proportion of proanthocyanins and anthocyanins determines the color of the seed coat.

With the rapid development of sequencing technology, multi-group approaches have become the primary means by which researchers explore previously uncharted areas of cellular biology. Metabolomics is used to determine the metabolic profile of crops under specific conditions and is considered a bridge between genotypes and phenotypes [18]. The transcriptome typically clarifies the regulatory mechanisms of a trait of interest by comprehensively analysing gene expression. The combined analysis of transcriptomics and metabolomics explores the causal relationship between genes and metabolites. Combined with the functional annotation of metabolic pathways and biological functional analysis, plant regulatory mechanisms can be systematically analyzed [19]. Multi-omics research, such as transcriptomics, proteomics and metabolomics, is widely performed on plant leaves [20,21] and seeds [6,22]. However, limited metabolomic research focuses on detecting metabolites in the soybean seed coat.

The soybean seed coat color is a main agronomic feature that determines seed quality. The seed coat color is closely related to appearance quality and nutritional value [23]. Compared with yellow seed coat soybean, the black seed coat soybean germplasm has a higher nutritional value in breeding programs [24]. Nevertheless, little is known about the metabolites in soybean seed coats of different colors within a wide range of soybean seed germplasm resources. Therefore, the purpose of this study was to detect and quantify the composition and content of flavonoids in five soybean germplasms with different seed coat colors via metabolome-based methods. The related genes controlling seed coat color were explored by transcriptome sequencing, and the candidate genes regulating the coloring mechanism of soybean seed coat were screened to provide important information regarding the genetic improvement of high-quality, nutritious soybean varieties.

## 2. Results

### 2.1. Metabolic Differences Among the Seed Coat of Soybean Germplasms

**Figure 1:**
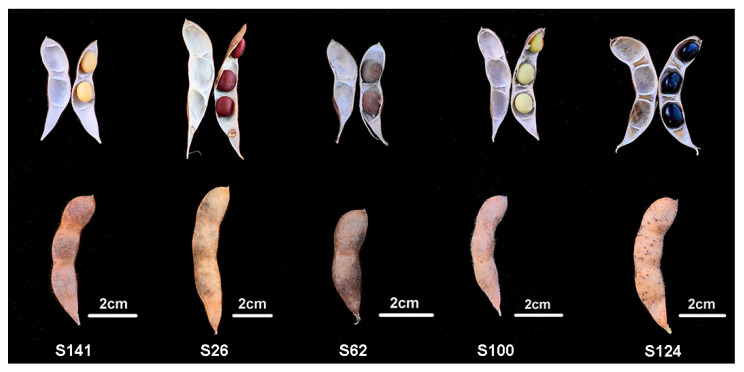
Phenotypes of soybean germplasms. S141: yellow seed coat; S26: red seed coat; S62: brown seed coat; S100: green seed coat; S124: black seed coat.

**Figure 2:**
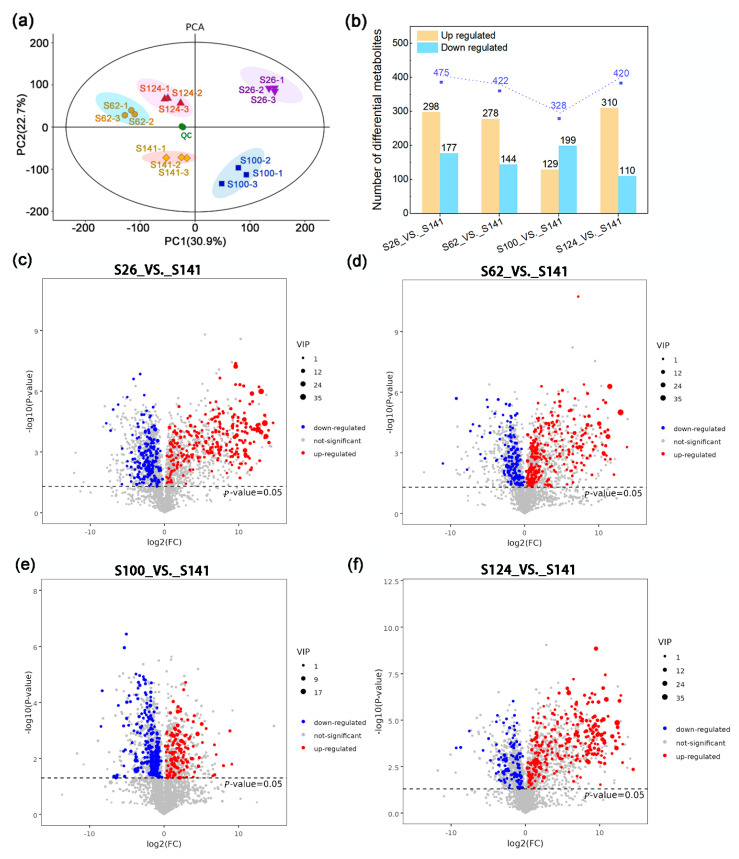
Differentially accumulated metabolites of the metabolome in soybeans with different seed coat colors. (**a**). Principal component analysis of the metabolites detected in the soybean seed coat using three biological replicates. (**b**). Numbers of differentially expressed metabolites among S26, S62, S100, and S124. (**c**–**f**). Volcano plots of differentially expressed metabolites among S26_vs._S141, S62_vs._S141, S100_vs._S141, and S124_vs._S141. Red dots indicate the upregulation of metabolites, blue dots indicate the downregulation of metabolites, and grey dots indicate that there is no significant difference in the metabolites.

Five soybean cultivars with different seed coat colors and phenotypes of soybean germplasms were analyzed, and they were found to be in the mature stage (Figure 1 and Table 1). The systematic metabolic profiling of soybean seed coats with different colors was performed. The yellow seed coat soybean S141 was used as the control, and the results for S26, S62, S100, and S124 were labelled as S26_vs._S141, S62_vs._S141, S100_vs._S141, and S124_vs._S141, respectively (Figure 2). The PCA results showed the excellent reproducibility of the sample data. The scattering patterns of the five soybean germplasms in the PCA plot showed evident differences among the metabolite profiles (Figure 2a). A total of 475, 422, 328, and 420 DAMs had differential expressions among S26_vs._S141, S62_vs._S141, S100_vs._S141, and S124_vs._S141, respectively (Figure 2b). In addition, 475 DAMs were screened out in S26_vs._S141, of which 298 were upregulated, and 177 were downregulated (Figure 2b). Moreover, 422 DAMs were screened out in S62_vs._S141, of which 278 were upregulated and 144 were downregulated (Figure 2b). A total of 328 DAMs were screened out in S100_vs._S141, of which 129 were upregulated and 199 were downregulated (Figure 2b). A total of 420 DAMs were screened out in S124_vs._S141, of which 310 were upregulated, and 110 were downregulated (Figure 2b). The DAMs caused by different seed coat colors were described by volcano plots in order to visually assess the overall distribution of DAMs in all comparison groups (Figure 2c–f). 

### 2.2. Analysis of the DAMs in the Flavonoid Biosynthetic Pathway

**Figure 3:**
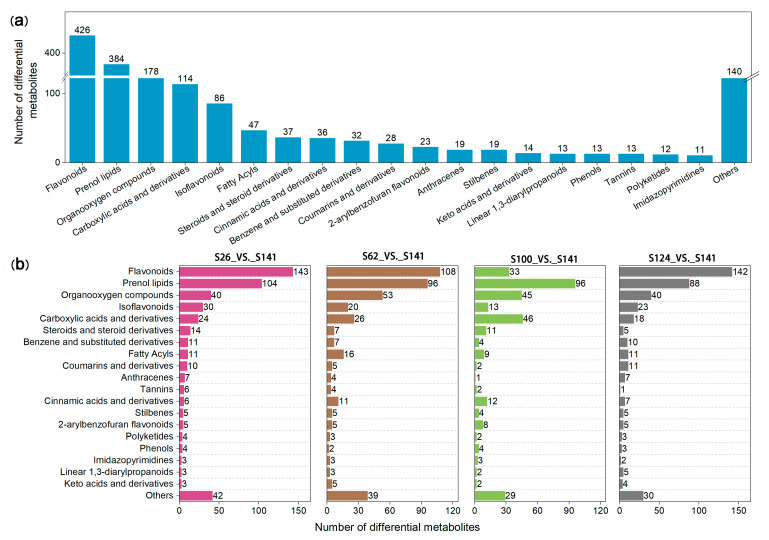
The classification of all the identified metabolites in the soybean seed coat. (**a**) classification of all the identified metabolites; (**b**) number of the DAMs.

A total of 1645 DAMs were detected in the seed coat of five soybean germplasms (Table S2). These DAMs were divided into 19 categories, and the remaining 140 DAMs were assigned to the “others” group (Figure 3a). The dominant DAMs were flavonoids, prenol lipids, organooxygen compounds, carboxylic acids and their derivatives, and isoflavonoids. These DAMs accounted for 72.2% of the total DAMs. In total, 426 flavonoids were identified from these four sample groups (Figure 3a). The annotation of these flavonoids is detailed in Table S2. The metabolomics data show that 143, 108, 33, and 142 flavonoids were identified among the S26_vs._S141, S62_vs._S141, S100_vs._S141, and S124_vs._S141 groups, respectively (Figure 3b). Compared with S141, 130, 93, 9, and 133 flavonoids were upregulated and 13, 15, 24, and 9 flavonoids were downregulated in S26, S62, S100, and S124, respectively (Table S3). The highest number of flavonoids was detected in the S26_vs._S141 group, demonstrating that soybeans with red seed coats exhibited a different metabolic profile than those with yellow seed coats. The lowest number of flavonoids was detected in the S100_vs._S141 group, indicating that soybeans with green seed coats exhibited a similar metabolic profile to those with yellow seed coats. In addition, a high number of flavonoids was detected in the S62_vs._S141 and S124_vs._S141 groups, indicating that soybeans with black and brown seed coats exhibited a different metabolic profile compared with soybeans with yellow seed coats (Figure 3b). Overall, our results showed the abundant diversity of metabolites in local soybean germplasm seed coats.

Based on the metabolome data, a total of 426 flavonoids, with significant differences among the S26_vs._S141, S62_vs._S141, S100_vs._S141, and S124_vs._S141 groups, were composed of 365 upregulated and 61 downregulated metabolites (Table S3). The number of differentially expressed flavonoids between the S26_vs._S141 and S124_vs._S141 groups was almost identical, with the accumulation of 143 and 142 flavonoids upregulated in the seed coat. In S26 and S124, 130 and 133 flavonoids, including quercetin and its derivatives, procyanidins, kaempferol and its derivatives, epicatechin, and epigallocatechin derivatives, were upregulated in the soybean seed coat. These flavonoids included nine procyanidins (procyanidin, procyanidin B1, procyanidin B2, procyanidin B3, procyanidin B6, procyanidin C1, proanthocyanidin A1, proanthocyanidin A2, and prodelphinidin B) and nine anthocyanins (Table S3). The anthocyanins included three cyanidin derivatives (cyanidin 3-galactoside, cyanidin 3-gentiobioside, cyanidin3-O-(6″-malonyl-arabinoside), three malvidin derivatives (malvidin 3-glucoside-4-vinylguaiacol, malvidin 3-glucoside-4-vinylphenol, malvidin 3-laminaribioside), peonidin-3-glucoside, petunidin 3-(6″-*p*-coumaryl-glucoside) 5-glucoside), and pelargonidin 3-rhamnoside 5-glucoside (Table S3). The high levels of flavonoids in soybean seed coats have long been reported to provide additional nutritional value to soybean food and feed [13], indicating that black and red seed coats of soybeans may have higher nutritional value.

Among these DAMs, five, three, one, and five types of anthocyanins were upregulated in S26, S62, S100, and S124, respectively, compared with S141, and these anthocyanins might be the key metabolites influencing seed coat coloration in soybean. Three kinds of anthocyanins, namely, cyanidin 3-O-(6″-malonyl-arabinoside), malvidin 3-laminaribioside, and malvidin 3-glucoside-4-vinylphenol, exhibited 2.97 × 10^13^-fold, 2.57 × 10^12^-fold, and 1.12 × 10^3^-fold, as well as 2.17 × 10^12^-fold, 5.40 × 10^12^-fold, and 1.00 × 10^3^-fold, increments in S26_vs._S141 and S124_vs._S141, respectively (Table S3). Proanthocyanidin A1 exhibited 2.0 × 10^13^-fold and 2.2 × 10^12^-fold increments in S26_vs._S141 and S124_vs._S141, respectively, which are greater increments than those of proanthocyanidin A2 and other procyanidin. Therefore, specific anthocyanins such as cyanidin 3-O-(6″-malonyl-arabinoside), malvidin 3-laminaribioside, malvidin 3-glucoside-4-vinylphenol, and proanthocyanidin A1 can serve as landmark metabolites of red and black seed coat soybeans. Notably, petunidin 3-(6″-*p*-coumaryl-glucoside) 5-glucoside, cyanidin 3-galactoside, astilbin, 3-galloylgallocatechin, and kaempferol 3-(2-*p*-coumaroylsophoroside) 7-glucoside accumulated massively in S124. The presence of these compounds was 1.29 × 10^6^–6.79 × 10^12^ times higher in S124 than in S141 (Table S3). Therefore, we speculated that these DAMs might be key metabolites affecting the black seed coat color of S124. Peonidin-3-glucoside was 4.99-, 1.3-, 2.58, and 1.84-fold lower in S26_vs._S141, S62_vs._S141, S100_vs._S141, and S124_vs._S141, respectively. By contrast, procyanidin, procyanidin B1, procyanidin B2, procyanidin B3, procyanidin B6, procyanidin C1, proanthocyanidin A1, proanthocyanidin A2, and prodelphinidin B were increased in S26_vs._S141, S62_vs._S141, and S124_vs._S141, respectively (Table S3). Previous studies have shown that the dark seed coat mainly contains anthocyanins and procyanidins, and the composition ratio and distribution of the two determine the coloring degree of the seed coat [24]. Similar results were also reported for cyanidin 3-O-glucoside in some plants with red flowers or fruit [25,26].

Three malvidin derivatives, one pelargonidin derivative, one petunidin derivative, and two cyanidin derivatives were accumulated in deep-color seed coat soybeans (red, brown, and black). Among these, cyanidin 3-O-(6″-malonyl-arabinoside) and malvidin 3-laminaribioside were the most abundant in S26 and S124, indicating that they were the primary sources of the red and black pigments. However, petunidin 3-(6″-*p*-coumaryl-glucoside) 5-glucoside was almost undetectable in S26, which indicated that petunidin 3-(6″-*p*-coumaryl-glucoside) 5-glucoside is the primary source of black phenotypes. Moreover, kaempferol and its derivatives, quercetin and its derivatives, and epicatechin were identified in S26_vs._S141. In particular, the presence of trifolin, quercetin, epicatechin-(4beta->8)-epigallocatechin 3-O-gallate isorhamnetin 3-(6″-malonylglucoside), and kaempferol 4′-glucoside 7-rhamnoside was 2.17 × 10^12^–2.97 × 10^13^ times higher in S26 than in S141 (Table S3), implying that these flavonoid compounds play essential roles in deepening the red color of soybean seed coats.

### 2.3. Transcriptomic Profiling of Soybean Seed Coat

**Figure 4:**
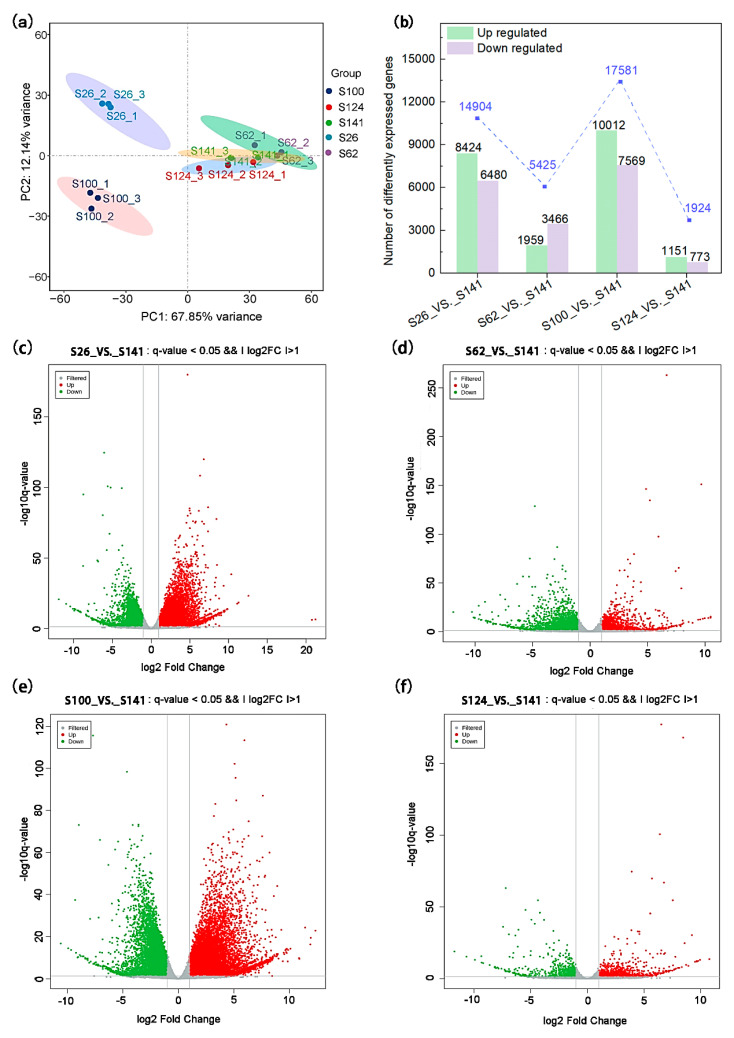
Analysis of transcriptomics data of soybeans with different seed coat colors. (**a**) Principal component analysis of the genes detected in the soybean seed coat with three biological replicates. (**b**) Number of differentially expressed genes among S26, S62, S100, and S124. (**c**–**f**). Volcano plots of differentially expressed genes among S26_vs._S141, S62_vs._S141, S100_vs._S141, and S124_vs._S141. Red dots indicate the upregulation of gene expression, green dots indicate the downregulation of gene expression, and grey dots indicate an absence of a significant difference in gene expression.

RNA-seq analysis was performed to investigate the transcriptome of soybean germplasms with different seed coat colors and to determine the molecular basis of the metabolic differences. In this study, we obtained 101.51 G of clean data, and the compelling data of each sample were distributed between 6.16 and 7.16 G. Moreover, the Q30 value (sequencing accuracy of 99.9%) was more than 93.30% (Table S4). The reads were aligned to the reference genome, and the ratio of successfully mapped reads ranged from 87.02% to 97.09%. PCA obtained excellent sample data reproducibility (Figure 4a). The PCA results showed large variations in S26_vs._S141 and S100_vs._S141, and less variation was observed in the S62_vs._S141 and S124_vs._S141 groups (Figure 4a). The DEGs, which emerged due to soybean germplasms with different seed coat colors, were described by volcano plots (Figure 4c–f). The comparison of S26_vs._S141 resulted in 14,904 DEGs, including 8424 upregulated and 6480 downregulated genes. The comparison of S62_vs._S141 resulted in 5425 DEGs, including 1959 upregulated and 3466 downregulated genes. The comparison of S100_vs._S141 resulted in 17,581 DEGs, including 10,012 upregulated and 7569 downregulated genes. The comparison of S124_vs._S141 resulted in 1924 DEGs, including 1151 upregulated and 773 downregulated genes (Figure 4b). The results indicated that these DEGs might play a key role in the expression of soybeans with different seed coat colors.

### 2.4. KEGG Pathway Enrichment Analysis of the Expected Changes in DAMs and DEGs

**Figure 5:**
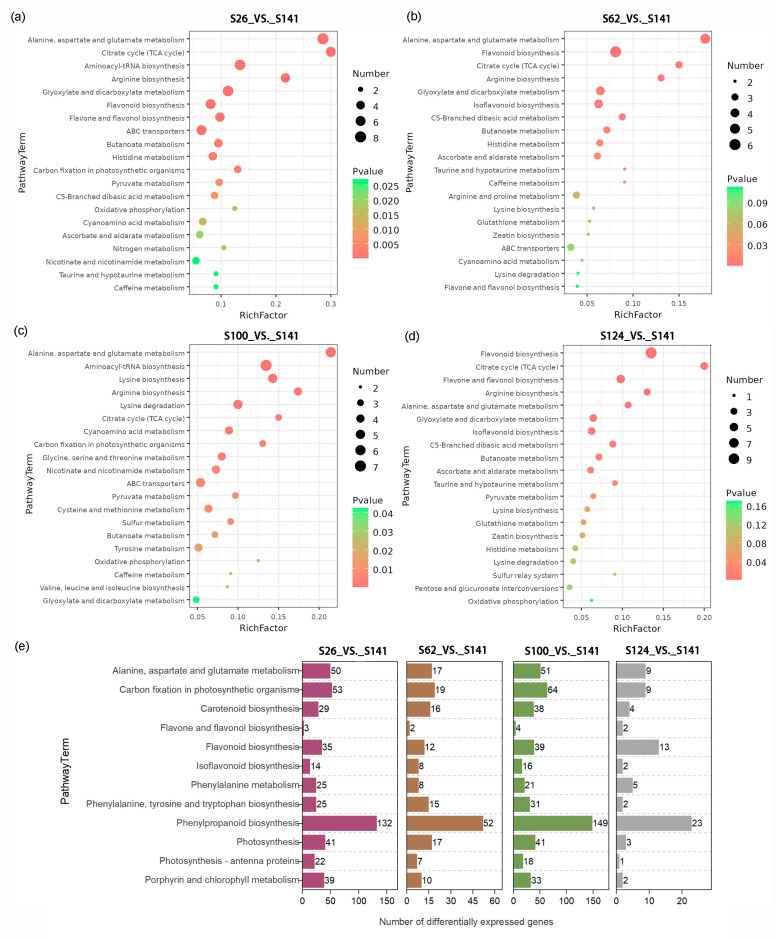
KEGG enrichment analysis of differentially accumulated metabolites and differentially expressed genes in soybeans with different seed coat colors. (**a**–**d**). KEGG enrichment analysis of differentially accumulated metabolites. The *x*- and *y*-axes represent the enrichment factor and pathway term, respectively. The colors and sizes of the dots represent the significance and number of metabolites, respectively. (**e**). KEGG enrichment analysis of differentially expressed genes.

All DAMs were mapped to the KEGG (Kyoto Encyclopedia of Genes and Genomes) database, and a total of 43, 41, 46, and 42 DAMs in the S26_vs._S141, S62_vs._S141, S100_vs._S141, and S124_vs._S141 groups were mapped to the KEGG pathways, respectively (Table S5). Similarly, KEGG enrichment analysis was conducted on the DEGs, and the results showed that a total of 3161, 1210, 3773, and 401 DEGs in the S26_vs._S141, S62_vs._S141, S100_vs._S141, and S124_vs._S141 groups were mapped to the KEGG pathway, respectively (Table S6). A large number of DAMs and DEGs were enriched into these pathways, which were associated with phenylpropanoid biosynthesis (gmx00940), flavonoid biosynthesis (gmx00941), flavone and flavonol biosynthesis (gmx00944), carotenoid biosynthesis (gmx00906), porphyrin and chlorophyll metabolism (gmx00860), and anthocyanin biosynthesis (gmx00942, Figure 5).

Mapping the differentially accumulated flavonoid metabolites among the five soybean germplasm seed coats showed that S26 had the highest relative content of all flavonoid metabolites (Table S5). Compared with S141, 10 compounds were detected that involved flavonoid biosynthesis, including quercetin, isoquercitrin, phloretin, 3,7-dimethylquercetin, luteoforol, catechin, phlorizin, quercetin 3-(6″-malonyl-glucoside), isowertin 2″-rhamnoside, and (+)-gallocatechin, which were significantly upregulated in S26 (Table S5). The results showed that there were significant differences in metabolites among the four groups with different seed coat colors, including 11 (10 upregulated and 1 downregulated), 9 (6 upregulated and 3 downregulated), 4 (1 upregulated and 3 downregulated), and 16 (13 upregulated and 3 downregulated) DAMs in the S26_vs._S141, S62_vs._S141, S100_vs._S141, and S124_vs._S141 groups, respectively (Table S5). Moreover, the presence of DAMs in the flavonoid biosynthetic pathways of the S26_vs._S141, S62_vs._S141, and S124_vs._S141 groups was significantly increased, indicating that these groups were related to the seed coat color changes of soybean, especially phloretin, luteoforol, catechin, and phlorizin (Table S5). Therefore, we hypothesized that those DAMs produced in the flavonoid biosynthetic pathways might be key metabolites in the deep-colored seed coat of soybean germplasm. Two flavonoids that comprised quercetin and isoquercitrin were identified from 40 flavonoid metabolites, and they were only found in S26 soybean. Quercetin accounted for the most significant proportion of upregulated metabolites, followed by isoquercitrin and phloretin (Table S5). This suggests that quercetin, isoquercitrin and phloretin may also play important roles in the formation of the red soybean seed coat color.

### 2.5. Candidate Genes Related to the Flavonoid Biosynthetic Pathway

**Figure 6:**
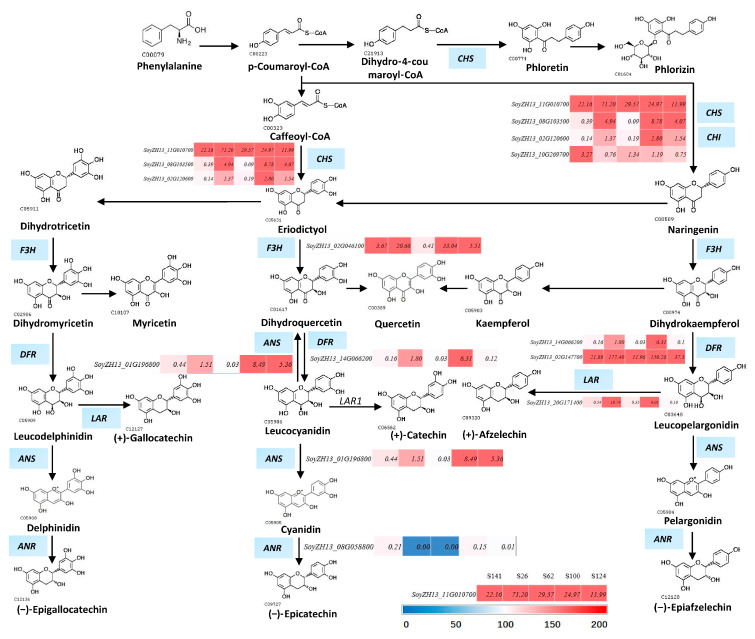
Flavonoid pathway in soybean with different seed coat colors. CHS: chalcone synthase; CHS6: chalcone synthase 6; CHS1: chalcone synthase 1; UGT88F4: UDP-glycosyltransferase 88F4; FTH: Naringenin,2-oxoglutarate 3-dioxygenase (fragment); DFR: bifunctional dihydroflavonol 4-reductase/flavanone 4-reductase; ANR: anthocyanidin reductase ((2S)-flavan-3-ol-forming), LAR: leucoanthocyanidin reductase. The colored rectangle represents the up- or downregulation of S141, S26, S62, S100, and S124, respectively, and the red rectangle represents a high FPKM value compared with the blue rectangle.

We annotated the functions of 13 DEGs based on the GO and KEGG databases to anchor the candidate genes for seed coat color in soybean. The results indicated that the genes *SoyZH13_11G010700 (CHS)*, *SoyZH13_08G103500 (CHS1)*, *SoyZH13_02G120600 (CHS6), SoyZH13_10G269700 (CHI1B2)*, *SoyZH13_02G046100 (FHT)*, *SoyZH13_14G066200 (DFR)*, *SoyZH13_02G147700 (DFR)*, *SoyZH13_20G171400 (LAR)*, *SoyZH13_01G196800 (ANT17)*, *SoyZH13_08G058800 (ANR)*, *SoyZH13_05G078700 (FLS)*, *SoyZH13_12G118300 (FG3)*, *SoyZH13_20G225300 (CHI2-A)*, and *SoyZH13_06G290900 (UGT88F4)* might be involved in the response to seed coat color in soybean (Figure 6). They were involved in the flavonoid biosynthetic pathway. Compared with S141, the genes *FG3*, *CHS*, *CHS1*, *CHS6*, *CHI2-A*, *UGT88F4*, *FHT*, *DFR*, *ANT17*, *LAR*, and *FLS* were upregulated in S26, whereas the gene *ANR* was downregulated. The genes *FG3* and *LAR* showed 78.61- and 18.8-fold increments in S26 compared with S141, whereas the gene *ANR* showed 0.04-fold degression (Table 2). The genes *FHT*, *ANT17*, *ANR*, *UGT88F4*, and *FLS* were downregulated in S62 compared with S141, whereas *CYP75B2* showed a 9.28-fold degression (Table 2). The expression levels of *CHS6*, *CHS1*, *UGT88F4*, *FHT*, *DFR*, *ANT17*, *LAR*, and *FLS* were upregulated in S100 compared with S141 (Table 2). *CHS6*, *CHS1*, and *ANT17* were upregulated in S124, whereas *CHS*, *ANR*, *LAR*, *UGT88F4*, and *FLS* were downregulated compared with S141. The genes *CHS6*, *CHS1*, *FG3*, and *ANT17* were significantly and highly expressed in S124 compared with S141, and their levels were 11.24-, 10.3-, 6.81-, and 12.17-fold higher than those in S141, respectively (Table 2).

**Figure 7:**
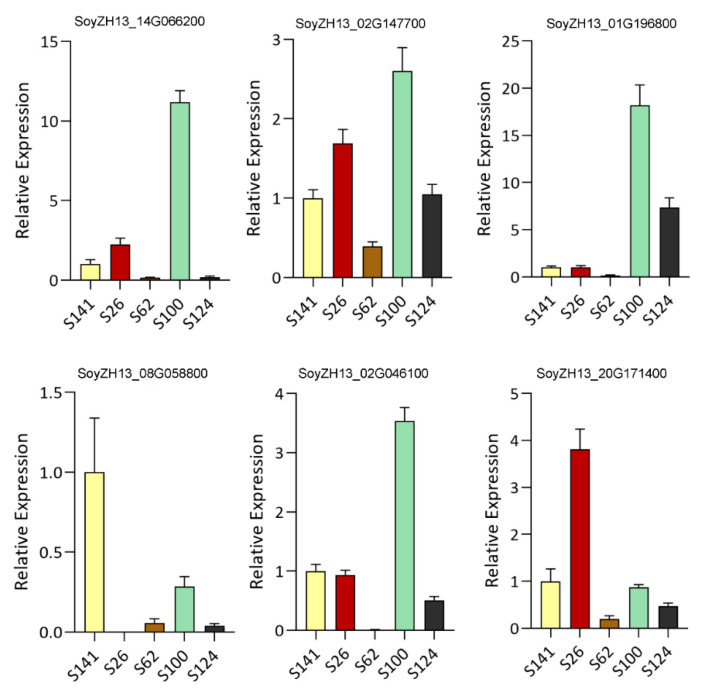
qRT-PCR detection of 6 key differentially expressed genes. The *x*-axis represents the relative expression of samples in qRT-PCR, and the *y*-axis represents the FPKM value in transcriptomics. The error bars indicate the SDs of three biological replicates. SoyZH13 14G066200 (*DFR*); SoyZH13 02G147700 (*DFR*); SoyZH13 01G196800 (*ANT17*); SoyZH13 08G058800 (*ANR*); SoyZH13 02G046100 (*FHT*); SoyZH13 20G171400 (*LAR*).

We randomly selected and measured six genes by qRT-PCR to verify the identified differentially expressed genes. These genes exhibited an expression pattern similar to the profiles of differentially expressed genes in soybean seed coats identified through RNA-seq analysis (Figure 7).

## 3. Discussion

### 3.1. Identification of Flavonoid Compounds in Soybean Seed Coat

The transcriptome and metabolome have enabled the development of effective methods to determine gene and metabolite components [27]. At present, few omics studies have been conducted on soybean seed coats. In this research, metabolic profiling and transcriptome analyses were used to explore the changes in relevant flavonoid compounds of soybean seed coats and their respective genes with different seed coat colors. Our results show that 1645 metabolites were identified in soybean seed coats using a non-targeted method. Compared with yellow seed coat soybean, more flavonoid metabolites were detected in red, brown, and black seed coat soybean, whereas fewer flavonoid metabolites were detected in green soybean seed coats in this research (Figure 3b). Consequently, the rich flavonoid metabolites identified in this research provide valuable reference information about soybeans with high nutritional values.

Flavonoids are natural phenolic secondary metabolites widely distributed in higher plants, including quercetin, kaempferol, flavonol, flavone, chalcones, anthoxanthins, and proanthocyanidin [9,10]. The phenylpropanoid biosynthetic pathway forms flavonoids, and they play major roles in various biological processes because of their effect on several characteristics of plant organs, imparting various colors, antioxidant properties, and nutritional value [11,12]. In plants, the accumulation of flavonoid compounds results in the different colors of plant tissues and is closely related to the types and amounts of flavonoids [7,10]. In our study, 426 flavonoids were detected in five soybeans with different seed coat colors. We found five flavonoids, including cyanidin 3-O-(6″-malonyl-arabinoside), proanthocyanidin A1, quercetin, kaempferol 4′-glucoside 7-rhamnoside, and kaempferol 3-rhamnoside 7-xyloside, to be enriched in the deep-colored seed coat of soybean germplasm. The content of these flavonoids was highest in red seed coats. We also found a higher content of kaempferol 3-(2-*p*-coumaroylsophoroside) 7-glucoside, cyanidin 3-O-(6″-malonyl-arabinoside), and proanthocyanidin A1 in black seed coats (Table S3). The chemical traits of anthocyanins have been widely investigated [10]. Anthocyanins and their contents are the key factors influencing the coloration of plant tissues, and they comprise pelargonidin, cyanidin, and delphinidin [28]. Cyanidin contributes to the red color of the soybean seed coat [29]. Our study detected cyanidin 3-O-(6″-malonyl-arabinoside) in the red seed coat. Compared with the yellow seed coat, the content of cyanidin 3-O-(6″-malonyl-arabinoside) in the red seed coat increased 2.97 × 10^13^-fold, indicating that cyanidin 3-O-(6″-malonyl-arabinoside) may play a key role in the coloration of the red seed coat of soybean (Table S3). This finding is similar to the results of Fu et al., who suggested that cyanidin 3-O-(6″-malonyl) glucoside is the major source of red phenotypes [12].

We found that the accumulation of flavonoids in the soybean seed coat of different colors was inconsistent. Interestingly, the flavonoids between the red and yellow seed coat of soybean showed that quercetin, quercetin 4′-glucoside, quercetin 3-beta-laminaribioside, and kaempferol contents were significantly higher in red seed coats than in yellow seed coats (Table S3). Some studies show that seed metabolites are directly related to seed quality and nutritional values and that the metabolites fluctuate greatly with seed coat color in a wide range of soybean seed germplasm resources [21]. The contents of isoflavones and the composition of fatty acids were affected by the seed coat color [30]. More in-depth studies of the seed coat metabolomes of red soybeans would help us to understand their unique metabolism and nutritional values better. 

Meanwhile, we found that the expression level of genes related to quercetin and kaempferol was increased, indicating that quercetin and kaempferol compounds were regulated at the transcriptional level. A few studies reported that peonidin is distributed in purple sweet potato and black grain, and it is a crucial flavonoid component that changes the color of the grain from green to black [9,28]. However, peonidin-3-glucoside is downregulated in red, brown, green, and black soybean seed coats among these flavones compared with yellow seed coat soybean. This result was different from a previous study on purple sweet potato and black grain, suggesting that this flavonoid metabolite expression level was related to the type and variety of plant tissue. 

### 3.2. DEGs Related to the Biosynthesis of Flavonoids in the Seed Coat of Soybean

The transcriptome is widely used to analyze the molecular mechanism of metabolite accumulation [31,32]. In this paper, we found that a large number of DEGs are enriched during phenylpropanoid biosynthesis, flavonoid biosynthesis, and anthocyanin biosynthesis in KEGG annotation, such as *CHS*, *FHT*, *ANT17*, *ANR*, *UGT88F4*, and *FLS*, and these genes exhibit differential expression (Table 2). *CHS6*, *CHS*, *CHS1*, *UGT88F4*, *FHT*, *DFR*, *ANT17*, *LAR*, *FLS*, *FG3*, and *CHI2-A* were significantly upregulated in S26_vs._S141, whereas *ANR* was significantly downregulated. Similar results were also exhibited in S100_vs._S141 (Table 2). The biosynthesis of flavonoids was significantly enriched in the transcriptome comparison of S26_vs._S141, S100_vs._S141, and S124_vs._S141 and was enriched in the corresponding metabolome comparisons of S26_vs._S141, S62_vs._S141, and S124_vs._S141 (Table 2 and Table S5). Although the biosynthesis of flavonoids was not significantly enriched in the transcriptome comparison of S62_vs._S141, it was enriched in the metabolome comparison of S62_vs._S141, indicating the presence of posttranscriptional regulatory activity, and similar results were reported in cassava flesh [18,33]. DEGs (*CHS1*, *UGT88F4*, *FHT*, *DFR*, *ANT17*, *LAR*, and *FLS*) in S62_vs._S141 showed a contrary expression pattern compared with those in S100_vs._S141. *UGT88F4*, *DFR*, *LAR*, and *FLS* in S124_vs._S141 also showed a contrary expression pattern compared with those in S100_vs._(Table 2). Flavonoids and DEGs were significantly enriched, and they showed a similar expression pattern in the transcriptome and metabolome comparisons of S26_vs._S141. These results suggest that soybean seed red coat pigmentation is potentially regulated at the transcriptional level.

Previous research reported that upregulating the expression level of *FLS* improved anthocyanin accumulation and changed the flower color of *P. suffruticosa* and *C. nitidissima* [12,34]. *CHS* and *FLS* play vital roles in the formation of the golden flowers of *C. nitidissima* [35]. When the expression of *ANR1* and *ANR2* was inhibited, the soybean seed coat showed a red-brown color [36]. In this study, the expression level of the *CHS* and *FLS* genes was upregulated (Table 2), which was in accordance with the significant increment in the content of cyanidin 3-O-(6″-malonyl-arabinoside) and malvidin 3-laminaribioside in S26_vs._S141. As shown in Table 2, comparison-based analysis revealed that the downregulation of genes encoding *UGT88F4*, *FLS*, *ANR* and *LAR* was found in S62_vs._S141 and S124_vs._S141, which was inconsistent with the significant increment in the content of pelargonidin 3-rhamnoside 5-glucoside, malvidin 3-glucoside-4-vinylphenol, cyanidin 3-O-(6″-malonyl-arabinoside), petunidin 3-(6″-*p*-coumaryl-glucoside) 5-glucoside, and malvidin 3-laminaribioside (Table 2). Anthocyanin biosynthesis involves multiple enzymes encoded by early biosynthesis genes (*CHS*, *CHI*) and anthocyanin-specific biosynthesis genes (*DFR*, *ANS*, and *UFGT*) [34]. In this study, the expression levels of *LAR*, *DFR*, *CHS6*, and *FLS* in S26 were higher than those in S141, whereas the transcriptional levels of *ANR* were lower than those of S141 (Table 2). ANR can convert anthocyanins into epicatechins and promote the biosynthesis of proanthocyanins [37]. A low level of expression of *ANR* genes leads to the abundance of anthocyanidins in the flavonoid biosynthesis of S26. We found that the cyanidin 3-gentiobioside content of S100 is considerably higher than that of S141. Thus, we speculated that the high expression of *DFR* in S100 can be related to the accumulation of cyanidin 3-gentiobioside. *FLS* encodes a flavonol synthase and can affect plant pigment, and anthocyanin content is reduced during *FLS* over-expression lines in *Arabidopsis*. This study found that an *FG3* gene was differentially expressed and upregulated 78.61-fold in the red seed coats. We deduced that the higher transcription level of *FG3* red seed coats may redirect the biosynthesis of cyanidin 3-O-(6′-malonyl-arabinoside) and proanthocyanidin A1. These results indicate that the genes mentioned earlier might play essential roles in the color diversity of soybean seed coats. We will see this in the next step of this research. 

## 4. Materials and Methods

### 4.1. Plant Materials and Experimental Site

Field experiments were conducted in 2021 at the Modern Agricultural Science and Technology Innovation Demonstration Park of Sichuan Academy of Agricultural Sciences in Xindu District, Sichuan, China (30°40′ N, 103°54′ E). The seed coat colors of the soybean germplasms “Huangdou” (S141), “Hongpixiangdou” (S26), “Bendihuangdou” (S62), “Bendiluhuangdou” (S100), and “Heipidadou” (S124) were yellow, red, brown, green, and black, respectively (Figure 1). A randomized block design was adopted. Soybean seeds were planted on 17 June 2021 and harvested on 25 October 2021. The row-to-row and plant-to-plant distances of soybeans were 50 and 20 cm, respectively. The fertilization scheme and field management followed Yang’s method [38]. At the R7 (beginning of maturity, one normal pod on the main stem has reached its mature pod color) stage of soybean growth, the seed coat of soybean germplasm was sampled and frozen in liquid nitrogen. The soybean seed coat samples were stored in a freezer at −80 °C until metabolome and transcriptome analyses. The analysis was performed with three biological replicates.

### 4.2. Metabolome Measurement

The metabolites in the soybean seed coat were detected by liquid chromatography–mass spectrometry (LC-MS). Using the method described by Xiao et al. [18], the metabolites were extracted, detected, identified, quantified, and analyzed by Shanghai Luming Biological Technology Co., Ltd. (Shanghai, China). 

Then, 80 mg of soybean seed coat powder was weighed, and 20 μL of internal standard (2-chloro-L-phenylalanine, 0.06 mg/mL; prepared in methanol), 1 mL of a methanol–water mixture (7/3, *v*/*v*), and two small steel balls were added. The mixture was precooled at −20 °C and then ground. Ultrasonic extraction was performed in an ice-water bath for 30 min, followed by overnight storage at −20 °C. The samples were centrifuged at 13,000 rpm and 4 °C for 10 min. A total of 150 µL of supernatant was collected using a syringe, filtered through a 0.22 μm microporous membrane, transferred to an LC injection vial, and stored at −80 °C until LC-MS analysis [39]. 

The extraction solution was analyzed using an ACQUITY UPLC I-Class plus machine (Waters Corporation, Milford, CT, USA) connected to a Q-Exactive mass spectrometer (Thermo Fisher Scientific, Waltham, MA, USA) operating with both positive and negative ion modes within an electrospray ionization source (ESI). The liquid chromatography conditions were as follows: an ACQUITY UPLC HSS T3 column (1.8 μm, 100 × 2.1 mm) was used. The mobile phase consisted of two components: solvent A (0.1% formic acid in water, *v*/*v*) and solvent B (0.1% formic acid in acetonitrile, *v*/*v*). Sample measurements were made using an elution, the gradient of which is shown in Table 3. The flow rate was maintained at 0.35 mL/min throughout the analysis. The injection volume was 2 μL, and the column temperature was set to 45 °C. Mass spectrometry data acquisition was performed in both positive and negative ion scanning modes. Mass spectrum parameters are shown in Table 4. 

The raw LC-MS data were processed using Progenesis QI V2.0 software (Waters Corporation, Milford, CT, USA). This involved baseline filtering, peak identification, integration, retention time correction, peak alignment, and normalization. Compounds were identified using the Human Metabolome Database (HDBM), Lipidmaps (V2.3) and a self-built database (Luming Biotech CO., Ltd., Shanghai, China). For the obtained data, we removed ion peaks with a missing value (0 value) > 50% in the group, replaced a 0 value with half of the minimum value, and screened qualitative compounds according to the qualitative result score. Compound identification was performed based on exact molecular weight matching in MS/MS (20 points), fragment matching in MS/MS (20 points), and isotope distribution matching (20 points), with a total score of 60 points. Compounds scoring below 36 points were excluded as inaccurate identifications. Quality control (QC) samples were prepared by mixing in equal volumes the extracts from all samples. Positive and negative ion data were combined into a data matrix for further analysis [40].

Principal component analysis (PCA) was performed in R to determine the relationships among the sample replicates. Orthogonal partial least-squares discriminant analysis (OPLS-DA) and partial least-squares discriminant analysis (PLS-DA) were used to distinguish the differentially accumulated metabolites (DAMs) among the groups. Model quality was evaluated using seven-fold cross-validation and 200 permutations of response testing. Metabolites with variable importance in projection (VIP) scores > 1 and a *p*-value < 0.05 were chosen as significant DAMs. Pathway analysis of DAMs was conducted using the KEGG database. Differential metabolites were further used for the enrichment analysis of the KEGG pathway (http://www.genome.jp/kegg/, accessed on 1 June 2023). Metabolomics data were deposited in the EMBL-EBI MetaboLights database (DOI: 10.1093/nar/gkad1045, PMID:37971328, accessed on 7 November 2024) with the identifier MTBLS11608.

### 4.3. Transcriptome Analysis

All freeze-dried soybean seed coats were ground by liquid nitrogen, and total RNA was extracted using the TRIzol reagent (Invitrogen, Carlsbad, CA, USA). RNA purity and quantification were evaluated using the NanoDrop 2000 spectrophotometer (Thermo Scientific, Waltham, MA, USA). RNA integrity was assessed using the Agilent 2100 Bioanalyzer (Agilent Technologies, Santa Clara, CA, USA). Then, the libraries were constructed using the VAHTS Universal V6 RNA-seq Library Prep Kit following the manufacturer’s instructions. Transcriptome sequencing and analysis were conducted by OE Biotech Co., Ltd. (Shanghai, China). The libraries were sequenced on an Illumina Novaseq 6000 platform, generating 150 bp paired-end reads. The clean reads were mapped to the reference genome using HISAT2. The FPKM of each gene was calculated, and the read counts of each gene were obtained using the HTSeq-count.

PCA was performed using R (v 3.2.0) to evaluate the biological duplication of samples. Differential expression analysis was performed using DESeq2. We set a Q value of <0.05 and a foldchange of >2 or <0.5 as the threshold for significantly and differentially expressed genes (DEGs). Based on the hypergeometric distribution, GO and KEGG pathway enrichment analysis of DEGs was performed to screen significantly enriched terms. Three biological replicates were tested for transcriptome sequencing. The raw sequence data reported in this paper were deposited in the Genome Sequence Archive [41] of the National Genomics Data Center, China National Center for Bioinformation/Beijing Institute of Genomics, Chinese Academy of Sciences (GSA: CRA014031), and are publicly accessible at https://ngdc.cncb.ac.cn/gsa (accessed on 5 March 2024). 

### 4.4. Quantitative Real-Time Polymerase Chain Reaction Analysis

Six genes were randomly selected and measured for quantitative real-time polymerase chain reaction. Total RNA was extracted using the TRIzol reagent (Invitrogen, Carlsbad, CA, USA). Gene-specific primers were designed using primer 5. The primers are listed in Table S1. Actin was used as the reference gene.

## 5. Conclusions

We assessed metabolomes and transcriptomes and compared DAMs and DEGs in soybeans with different seed coat colors. In this study, soybeans with different seed coat colors exhibited distinct metabolic profile. Compared with yellow seed coat soybean, more flavonoid metabolites were detected in red, brown, and black seed coats, whereas less flavonoid metabolites were detected in green seed coat, especially procyanidins, procyanidin, malvidin and its derivatives, petunidin and its derivatives, and cyanidin and its derivatives. Cyanidin 3-O-(6″-malonyl-arabinoside), malvidin 3-laminaribioside, and petunidin 3-(6″-*p*-coumaryl-glucoside) 5-glucoside were the main anthocyanin components in the soybean seed coat. In addition, *CHS*, *CHI*, *DFR*, *FG3*, *ANR*, *FLS*, *LAR*, *UGT88F4*, and *UGT88F5* were identified as candidate genes contributing to the color diversity of soybean seed coats. These results broaden the knowledge of the metabolic and transcriptomic changes in soybean seed coat caused by color changes and provide new insights into the development of bioactive substances from soybean seed coats.

## Figures and Tables

**Table 1:** General information on soybean germplasms.

Code	Name	Seed Coat Color	100-Seed Weight (g)
S141	Huangdou	Yellow	16.4
S26	Hongpixiangdou	Red	36.8
S62	Bendihuangdou	Brown	19.7
S100	Bendilvhuangdou	Green	20.5
S124	Heipidadou	Black	20.7

**Table 2:** DEGs related to the flavonoid biosynthetic pathway.

	S26_vs._S141			S62_vs._S141			S100_vs._S141			S124_vs._S141		
	Fold Change	*p*-Value	Regu-lation	Fold Change	*p*-Value	Regu-lation	Fold Change	*p*-Value	Regu-lation	Fold Change	*p*-Value	Regu lation
SoyZH13_02G120600(CHS6)	8.65	0.010	up	1.54	0.660	up	17.30	0.000	up	11.24	0.000	up
SoyZH13_11G010700(CHS)	2.83	0.000	up	1.36	0.580	up	0.95	0.860	down	0.54	0.010	down
SoyZH13_08G103500(CHS1)	10.75	0.000	up	0.23	0.060	down	18.76	0.000	up	10.30	0.000	up
SoyZH13_06G290900(UGT88F4)	1.51	0.014	up	0.41	0.000	down	1.75	0.000	up	0.50	0.001	down
SoyZH13_02G046100(FHT)	5.35	0.000	up	0.12	0.000	down	7.71	0.000	up	1.51	0.260	up
SoyZH13_14G066200(DFR)	10.47	0.000	up	0.21	0.200	down	33.06	0.000	up	0.72	0.730	down
SoyZH13_02G147700(DFR)	7.51	0.000	up	0.57	0.220	down	5.87	0.000	up	1.71	0.100	up
SoyZH13_01G196800(ANT17)	3.17	0.000	up	0.08	0.010	down	16.61	0.000	up	12.17	0.000	up
SoyZH13_08G058800(ANR)	0.04	0.010	down	0.05	0.030	down	0.63	0.550	down	0.08	0.030	down
SoyZH13_20G171400(LAR)	18.82	0.000	up	0.68	0.580	down	9.39	0.000	up	0.19	0.030	down
SoyZH13_05G078700(FLS)	7.76	0.000	up	0.26	0.020	down	4.39	0.000	up	0.05	0.000	down
SoyZH13_12G118300(FG3)	78.61	0.000	up				26.24	0.000	up	6.81	0.000	up
SoyZH13_20G225300(CHI2-A)	7.42	0.000	up				7.42	0.000	up			
SoyZH13_05G020900(CYP75B2)				9.275	0.002	up						
SoyZH13_12G061300(CYP93B1)							21.265	0.001	up			

CHS6: chalcone synthase 6; CHS: chalcone synthase; CHS1: chalcone synthase 1; UGT88F4: UDP-glycosyltransferase 88F4; FTH: Naringenin,2-oxoglutarate 3-dioxygenase (fragment); DFR: bifunctional dihydroflavonol 4-reductase/flavanone 4-reductase; ANT17: leucoanthocyanidin dioxygenase; ANR: anthocyanidin reductase ((2S)-flavan-3-ol-forming); LAR: leucoanthocyanidin reductase; FLS: flavonol synthase/flavanone 3-hydroxylase; FG3: UDP-glycosyltransferase 79B30; CHI2-A: chalcone–flavonone isomerase 2-A; CYP75B2: Flavonoid 3′-monooxygenase; CYP93B1: licodione synthase.

**Table 3:** The elution gradient.

Time (min)	A%	B%
0.01	95	5
2	95	5
4	70	30
8	50	50
10	20	80
14	0	100
15	0	100
15.1	95	5
16	95	5

A: 0.1% formic acid in water, *v*/*v*; B: 0.1% formic acid in acetonitrile, *v*/*v*.

**Table 4:** Mass spectrum parameters.

Parameters	Positive Ion Mode	Negative Ion Mode
Mass scan range (*m*/*z*)	100–1200	100–1200
Resolution (full scan)	70,000	70,000
Resolution (HCD MS/MS scans)	17,500	17,500
Spray voltage (V)	3800	−3000
Sheath gas flow rate (Arb)	40	35
Aux gas flow rate (Arb)	10	8
Capillary temperature (°C)	320	320
Auxiliary gas heater temperature (°C)	350	350
S-lens radio frequency (RF) level	50	50

## Data Availability

All data are fully available without restriction.

## Supplementary Materials

The following supporting information can be downloaded at: https://www.mdpi.com/article/10.3390/ijms26010294/s1.
